# Quality of family planning services in Mexico: The perspective of demand

**DOI:** 10.1371/journal.pone.0210319

**Published:** 2019-01-30

**Authors:** Pilar Torres-Pereda, Ileana B. Heredia-Pi, Midiam Ibáñez-Cuevas, Leticia Ávila-Burgos

**Affiliations:** Center for Health Systems Research, National Institute of Public Health Mexico, Cuernavaca Morelos, México; Brown University, UNITED STATES

## Abstract

**Introduction:**

Family planning (FP) is one of the key services provided by health care systems. Extending beyond matters of sexual and reproductive health, its area of influence impacts directly on the development of individuals and nations. After 60 years of intense FP activities in Mexico, and in light of recent restructuring of health service supply and financing, services need to be assessed from a user perspective.

**Objective:**

Based on a comprehensive conceptual framework, this article assesses the quality of the FP services provided by the Mexican Ministry of Health (MoH). Analysis considers not only accessibility and availability but also the users’ perceptions of the care process, particularly as regards the interpersonal relations they experience with staff and the type of information they are provided.

**Material and methods:**

This study used a descriptive, qualitative design based on maximum variation sampling in six Mexican states. It included visits to 12 clinics in urban and rural areas. Thematic analysis was performed on 86 semi-structured interviews administered to FP service users.

**Results:**

While access was described by users as “easy,” their experiences revealed normalized barriers. One of our key findings referred to inverse availability, meaning that the contraceptive methods available were generally not the ones preferred by users, with their selection therefore being shaped by shortage of supplies. Challenges included disrespect for the free choice of FP users and coercion during consultations for contraception post obstetric event. Finally, information provided to users left considerable room for improvement.

**Conclusions:**

After six decades of FP service supply, results indicate a series of quality issues that may lie at the heart of the unmet demand reported in the literature. Based on a comprehensive conceptual scheme, the present study analyzes the quality of services, highlighting areas for improvement that should be considered by the MoH in future efforts.

## Introduction

Family planning (FP) represents a set of activities, procedures and interventions that provide the population with counseling, health education and modern contraceptive methods (CMs) in order that individuals may exercise their right to decide freely and responsibly whether to have children and, if so, the number and appropriate timing of their children.[1] Numerous international organizations and initiatives have promoted the involvement of governments in ensuring universal access to effective and quality FP services as a right of women and girls that is essential for leading a healthy life. Salient among these organizations are the World Health Organization (WHO) and the United Nations Population Fund (UNFPA). Additionally, initiatives such as the Sustainable Development Goals (SDGs) and the Global Strategy for Women’s, Children’s and Adolescents’ Health have championed the causes of FP through a universal call for action from the public sector. International recommendations have placed particular emphasis on improving equality and quality in service provision and on providing the most vulnerable population groups with greater access to FP care, as they are the ones who encounter the largest barriers to these services [2,3].

The numerous benefits of FP have an impact on health and development. For instance, by making it possible for women to decide when to become pregnant and how to space their pregnancies as desired, FP contributes to the *prevention of pregnancy-related health risks*. Particularly among young people, the ability to postpone pregnancy and avoid unplanned births lowers the number of unsafe abortions and abates maternal and child mortality [4,5]. Other benefits of effective FP programs include the *ability to continue schooling*. FP affords individuals the opportunity not only to make informed decisions on sexual and reproductive health matters, but also to extend their education and become more competitive in the labor market. The consequences of a truncated educational experience are harmful for the adolescent as well as for her children, her family and her community [4–6].

Mexico has consolidated a consistent trajectory of FP services since the 1970s, with accomplishments including a reduction in fertility from seven children per woman in 1960 to 2.21 in 2014. Progress has been linked primarily to promotional efforts and a consequent gradual increase in the use of CMs by people of reproductive age [7,8], particularly by those belonging to population groups lacking access to Health Care protection and residing in rural areas. In 2001, the government implemented the Fair Start in Life (*Arranque Parejo en la Vida*) Program with the aim of improving access to skilled birth care and strengthening FP services, particularly in those rural areas with the highest rates of maternal mortality [9,10].

In 2003, the government introduced the most important health care reform in the country as part of its efforts to expand health care coverage for the population without Health Care protection: the unemployed, self-employed and those engaged in agricultural work. It established the System of Social Protection in Health (*Sistema de Protección Social en Salud*) including its key component, the Popular Health Insurance (*Seguro Popular*, *SP*), a voluntary insurance scheme available only to beneficiaries, with enrollment free of charge [11,12]. Since its inception, the *SP* has offered its members a package of health interventions (currently 284) administered by the state-level MoH. By 2016, *SP* beneficiaries had reached 60.6 million, or 50.4% of the Mexican population [13–15]. In an attempt to meet the United Nations Millennium Development Goals, the government also integrated the 2003 health reform with policies aimed at improving maternal health and contraceptive coverage.

Specifically, with regard to FP, in 2014, the federal MoH restructured the CM purchase mechanisms, shifting this function from the state to the federal MoH level. The aim of this revision was to optimize the use of resources, reduce the price of inputs through the centralized acquisition of large volumes and, as a result, to increase CM supply and national FP program coverage. That same year, public investment in CM purchases totaled 974.1 million pesos, double the amount expended the year before [16,17].

Aligning maternal health and FP policies with the health care reform allowed for lowering financial barriers and improving access to FP services, particularly for SP beneficiaries [9,10]. As a result, contraceptive coverage grew from 70.9% in 2006 to 72.3% in 2009, remaining stable from 2009 to 2014 [18]. Condom use in adolescents increased from 31.8% in 2006 [19] to 47.8% in 2012 but has nonetheless failed to meet the 70% goal set by the health authorities [8]. The number of children considered ideal by women in this age group (15–49 years) dropped from 2.9 in 1997 to 2.6 in 2014. Finally, usage of CMs in this age group increased from 49.7% in 2009 to 51.6% in 2014 [7].

The expansion of reproductive health services resulted in an 82% increase in government expenditures for relevant programs from 2003 to 2015 [20], most of it attributable to greater spending by the institutions serving SP beneficiaries (342%).

In particular, expenditures on FP services for SP beneficiaries jumped 651%—primarily in response to the objective of reinforcing strategies to reduce adolescent pregnancies in 2014–2015 [20, 21]. As a consequence of efforts to improve FP coverage, the number of active users in the country grew considerably between 2013 and 2015; their specific proportions by type of method were 68.5% for transdermal patches, 63.6% for medicated intrauterine contraceptive devices-IUDs and 59.7% for subdermic implants [16].

Notwithstanding the preceding advances towards more effective FP services in Mexico, gaps still prevail with regard to access and development, particularly among residents of highly and very highly marginalized communities with rural and indigenous populations [8]. In 2012, coverage for contraception post obstetric events (CPOEs)—a strategy used by women to postpone or terminate their reproductive lives by adopting a temporary or permanent contraceptive method within 40 days following an obstetric event—[22] stood at 57.4% nationally but dropped to 50.1% in rural dwellers, 49.6% in indigenous women, and 47.9% in adolescents [23]. Specifically, regarding the access gaps in adolescents, in 2009, over 30% of married women between the ages of 15 and 19 experienced an unmet demand for contraception [24].

In addition to narrowing the gap in coverage, it is essential to improve the quality of care. Evidence suggests that providing quality FP services is conducive to greater utilization of these services and increased use of contraception [25]. Various studies have documented the persistence of problems concerning quality of care. In 2016, a study of Mexico evaluated the technical and interpersonal dimensions of quality. The first dimension was divided into three categories concerning whether or not users were offered information about (1) the variety of existing CMs; (2) the effects of each method; and (3) the possibility of returning for assistance. The second was divided into two categories related to whether or not users were offered (1) sufficient time during consultations to fully address the information they required, and (2) sufficient information to completely dispel any doubts regarding the chosen method. The sample of users was divided into three age groups: 15–19, 20–24 and 25–29 years old. The results of the study revealed gaps regarding the perceived quality of care among users from the different age groups: positive responses on all five quality aspects were obtained from 61% of the first (adolescents), 65% of the second and 67% of the third age group [6].

Given the efforts deployed by the Mexican government, particularly the health sector, to increase funding and access to FP services in Mexico, it is relevant to identify and understand the barriers and facilitators experienced by users as they navigate through the health care process to access and benefit from quality FP services. It is important to investigate how users perceive their care, for instance, in relation to how they are treated. This would elucidate the extent to which they are satisfied with their services and highlight areas of opportunity for improvement. The short-term effects of actions based on this evidence would include greater usage and improved health outcomes [26–28].

Considering the increased availability of resources for improving FP service provision and the need to incorporate the views of users in order to enhance quality, the present study sought to describe the perception of users as to the quality of care offered by the Mexican FP services. Special emphasis was placed on the following quality components: access, availability, interpersonal relations and information given to clients. Analysis encompassed FP services provided by the MoH in six states.

### Conceptual framework

Different conceptual frameworks have been proposed for evaluating FP service quality. In 1990, Bruce and Jain [26, 29–31] recommended a user perspective to evaluate the quality of FP services and CM supply based on the following criteria: (1) choice of method, (2) information provided, (3) technical competency, (4) interpersonal relations, (5) follow-up and continuity mechanisms, and (6) an adequate constellation of services. Evidence has shown that improving these dimensions redounds to greater use of services and, in turn, to improved outcomes [26, 29–31]. These criteria can be framed within the three classical quality dimensions proposed by Donabedian: program structure, service processes and outcome of care in terms of user knowledge, behavior and satisfaction [29,32,33]. While Bruce-Jain and Donabedian converge in their focus on user satisfaction, the former consider different FP dimensions and highlight the importance of establishing mechanisms to ensure follow-up and continuity in care.

In 1996, Creel et al. proposed a different conceptual framework for evaluating the quality of FP services. They considered ten dimensions of quality based on demand: (1) availability of CMs and possibility of choice; (2) absence of medical-bureaucratic hurdles; (3) an appealing physical setting that inspires confidence; (4) continuity in care; (5) assorted service offerings; (6) competent staff; (7) adequate schedules and waiting times; (8) affordability; (9) effective counseling; and (10) friendly interpersonal relations. While Creel et al. shared numerous elements with Bruce and Jain, the first added two new components: service accessibility and acceptability [26].

S1 Fig offers a full picture of the three models proposed by Bruce-Jain, Donabedian and Creel for evaluating the dimensions of quality in reproductive health services. Based on this conceptual framework, analyzing the quality of services from the perspective of those who demand service sheds light on the opinions of users as influenced by their personal values, experiences and concepts regarding the role of health services.

It is important to bear in mind that the components of quality are interrelated; for instance, free choice is related to the availability and variety of CMs. In other words, the capacity of a user to make an appropriate decision can be transferred to the possibility of choosing a CM of his or her preference [34]. CM availability at health facilities has been identified as a factor with one of the highest predictive values for user satisfaction—higher than that of other components, or dimensions, of quality [34]. The interpersonal relations expressed in user-provider interactions also play a decisive role in perceived quality. Studies have demonstrated that women seeking free or subsidized services from the public sector have experienced mistreatment, disrespect and even physical abuse [34]. These situations limit the capacity of users to demand quality health services and are often related to a differential treatment from health providers based on age, social class and economic/cultural status [34]. A study in Uganda [35] demonstrated that the perceptions of providers on the payment capacity of users influenced the care they offered, even determining the type of CM they prescribed. Finally, correct information provided to users as part of their FP services is relevant in that it affects their capacity and disposition to use certain methods [34].

While valuable contributions have been made to knowledge on these dimensions by previous studies [6] these analyses have largely been quantitative. Strategies aspiring to upgrade the quality of FP care would achieve more targeted efforts by complementing actions aimed at improving the behavior and infrastructure of service providers with qualitative evidence. The experience of users and the perspective of service demand play a highly relevant role in the quest for better care. Weight needs to be accorded to the opinions and necessities of users, particularly as regards the selection of contraceptives, the availability of preferred methods and the establishment of a respectful and private environment [26].

In light of the foregoing, this study used a qualitative approach to ascertaining the opinions of FP service users regarding four quality-related elements: (a) the presence and identification of barriers to accessing services; (b) the availability of CMs, with special emphasis on those preferred by users; (c) provider-user interpersonal relations; and (d) the type and quality of the information provided.

## Materials and methods

### Study design

Based on a descriptive, qualitative design, our study was conducted in public health facilities belonging to the MoH. We analyzed service quality specifically with regard to access, availability, interpersonal relations and information provided to clients, as perceived by users throughout their experience in the FP care process.

### Sample selection

#### Selection of health care facilities

Maximum variation sampling was performed in two stages. We first selected six states in the different cultural and socioeconomic regions of Mexico [36]: Nuevo Leon and Coahuila in the north; Mexico State and Morelos in the center; and Veracruz and Campeche in the south. We then selected two facilities per state—one in an urban and another in a rural setting. The aim was to ensure that analysis considered the differences resulting from the marginalized conditions in which rural–as opposed to urban—health services normally operate. All the facilities selected offered outpatient care to SP beneficiaries [37] and were situated in areas inhabited by mestizo populations (mixed racial or people of blended descent). We only sample mestizo populations because indigenous people may not speak Spanish. During the second sampling stage, we selected users of reproductive and maternal health services in each facility.

#### Selecting interviewees

The following inclusion criteria were adopted to select FP service users at each facility: (a) women and men >18 years of age scheduled for an appointment; (b) women (or their partners) >18 years of age scheduled for counseling on puerperium care or contraception; in both cases, only subjects who expressed their willingness to participate in the study were included in our sample.

The number of interviews was determined according to the theoretical saturation technique, as described by Bertaux [38]. We invited at least seven users from each state to participate in the study and added as many more as necessary to reach the data saturation point. Although the literature does not provide a specific number of cases for saturation [39], we chose a minimum of seven interviewees by state given the need to analyze at least three individuals regularly using FP services, three seeking CPOE consultation and one more to increase the number of observations. In line with other authors [40], we considered that reaching the theoretical saturation point by state would require at least three interviews by theme (contraception and CPOE); the additional case was incorporated with the view of reinforcing the accumulation of evidence across our sample [39].

The intention was to administer at least 42 interviews in order to accumulate as much evidence as possible at three different levels—health care facility, state and national—and achieve saturation and richness in our qualitative data [39]. With this as our goal in regard to minimum sample size, we included additional observations in the final study sample–as many as the field team could manage during the fieldwork periods assigned to each state.

### Field staff

Our field staff was comprised of two female coordinators and five interviewers (four female and one male). The interviewers were knowledgeable about FP matters, educated in social sciences and public health and widely experienced in the use of qualitative tools such as interviews. The field staff had sustained no previous relations with the interviewees and were not personnel from the clinics, but rather independent interviewers hired to assist the research team. They introduced themselves to the patients as researchers from the National Institute of Public Health. Prior to kickoff, the field staff received one week of training sessions on themes related to the research questions, the interview guide, effective interviewing and ethical behavior during fieldwork.

### Recruiting and data collection

Users were contacted at the selected primary-care facilities. The field team reviewed the appointment lists daily and identified users requiring FP services and/or follow-up care for puerperium or CPOE purposes; they had no access to their medical records. Every day during the fieldwork period, the field staff invited all the FP users on the facilities’ waiting lists to participate in the study. Listed individuals were approached in the waiting room without offering any participation incentive whatsoever. Those who agreed to participate were taken to a private area where their oral informed consent and permission to tape record the interviews were obtained. Very few users declined our invitation, all of them adducing lack of time. The interview length time averaged 30 minutes and ranged from 16 to 58 minutes. All the interviews were conducted in Spanish and were listened to only by the researchers.

### Instruments

We used a guide for semi-structured interviews (S1 and S2 Files). The questions explored general socio-demographic data (age, sex, marital status, educational level and occupation) but focused on the research themes: (a) reasons for seeking FP services; (b) experiences while accessing FP services; (c) experiences related to CMs—specifically access, availability, free choice, and previous as well as current use—whether at the primary-care facility where the interview took place or at another MoH facility; and (d) perceived treatment and interpersonal relations at the facility. The guide was first piloted among subjects with characteristics similar to those of the target population with the objective of testing the questions and enhancing the internal validity of the instrument. Notes taken during each interview were registered in a field journal.

### Analysis

Data collected were emptied into a response matrix. The response matrix is a table containing two or more cross-classified dimensions, variables or concepts [41,42]. We transcribed the discourses of our interviewees verbatim in the results matrix and captured the data using a line for each respondent and a column for each code or analytical category. To build the matrix, we used the elements explored during the semi-structured interviews as *a-priori* codes and added emerging codes as needed. Verbatim fragments of the discourses of users were keyed in and brief analytical notes annexed; the socio-demographic characteristics used to analyze the data were also entered. We used cross coding to ensure that the matrix included standardized codes; that is, all the members of the research team coded the same interview and incorporated the data from that interview into the matrix. Comparing and contrasting among team members allowed for standardizing the data captured under each code, thus optimizing the quality of data capture and ensuring reliability.

We then performed thematic analysis [43,44] by searching the analytical categories, or codes, for themes that could assist in understanding the phenomenon under study. We specifically sought categories that contained detailed explanations related to our research questions. Two parallel rounds of data readings were carried out by two members of the research team. A triangulation exercise was then undertaken to identify findings; these were examined in the light of our research questions and defined as themes that co-occurred in the analyses of both researchers. The credibility of our findings was thus ensured through the systematic and rigorous process of interpretive triangulation [45]. Our theoretical findings were based on detailed and sound information; they captured the experiences of respondents and provided an understanding of their thought patterns and interactions. These findings can therefore be transfered to other subjects presenting equivalent characteristics and seeking CPOE services at facilities similar to those analyzed. [46].

Our results are presented below in the form of verbatim fragments from the interviews recorded at the participating FP service facilities. In order to contextualize the voices in the users’ testimonies, the following labels were established: (1) state (Nuevo Leon, Coahuila, Mexico State, Morelos, Campeche and Veracruz); (2) sex (female and male); (3) age at the time of the interview (e.g., 42 years old); (4) marital status (married, single, free union, divorced, and widowed); (5) completed educational level (none, elementary school, middle school, high school, bachelor’s degree, and beyond); (6) occupation (housewife, employee, student and other); (7) CM being used (e.g., condom, hormonal, IUD, and bilateral tubal ligation-BTL); and (8) ID number ascribed to the interview (e.g., E14). All data were analyzed in Spanish; the English translation of this article was performed by a professional translator specializing in public health and sexual and reproductive health.

### Mechanisms for ensuring validity and rigor

The credibility and consistency of our data were ensured through interpretive triangulation, which involves the participation of several researchers in the analysis and interpretation of data [47]. The purpose of this analytical strategy was to achieve transferability of our findings based on theoretical saturation sampling [39]. Confirmability and validity of our data were established by comparing and contrasting the themes of our findings and concepts analyzed against the literature. We also held critical reflection sessions among team members in order to ensure agreement on the research problem, theory and methodology [48].

### Ethical aspects

In conformity with the ethical principles of the Helsinki Declaration for medical research involving human subjects, we provided an oral informed consent form to all potential interviewees. Those who agreed to participate were asked for their authorization to tape record their interviews. The study was approved by the Ethics and Research Committees of the National Institute of Public Health in Mexico.

## Results

The results in this article describe the perceptions of users in six Mexican states regarding four aspects of FP care: access, availability, interpersonal relations and information given to clients.

A total of 86 semi-structured interviews were administered to female and male users of selected health care facilities in six states: 16 in Nuevo Leon, 16 in Coahuila, 20 in Mexico State, 19 in Morelos, 9 in Veracruz, and 6 in Campeche. The sample size was smaller in Campeche because our field visits coincided with a shortage in the supply of contraceptives which caused a reduction in the attendance of FP users at participating facilities. The same situation was observed, although to a lesser extent, in the other states. Male respondents are not included in this analysis.

Table 1 shows the characteristics of the users interviewed: 58% lived in rural areas and were 25.9 years old on average. The majority (95%) were women who were married or living in union (98%), had completed middle school or less (64%) and worked in the home (73%). It is noteworthy that in a sample of 86 users, only four were men. This was the total number of male users found in the facilities during normal service hours.

**Table 1:** Characteristics of the population interviewed.

Users			
Age	Average (years)	25.9	
Variable	Characteristic	N	%
Age groups	15–19	19	0.23
	20–24	21	0.24
	25–29	25	0.30
	30–34	9	0.10
	35–39	9	0.10
	40–44	2	0.02
	45–49	1	0.01
	Total	86	1
Sex	Women	82	0.95
	Men	4	0.05
	Total	86	1
Marital status	Married	36	0.42
	Single	10	0.12
	Free union	38	0.44
	Divorced	1	0.01
	Widowed	1	0.01
	Total	86	1
Educational level	Elementary school	21	0.24
	Middle school	34	0.4
	High school	25	0.29
	Bachelor’s degree	5	0.06
	No schooling	1	0.01
	Total	86	1
Main occupation	Home	63	0.74
	Remunerated job	19	0.22
	Unemployed	2	0.02
	Student	2	0.02
	Total	86	1
Area of residence	Rural	50	0.58
	Urban	36	0.42
	Total	86	1

Source: prepared by the author based on the information collected during the interviews

### Access

Access to health services is the gateway to FP. Participants had generalized access to FP services and CMs. While they classified their access as easy, barriers clearly existed—albeit systematically indicated and normalized as part of the health care process. Only a few respondents defined their access as difficult. The testimonies compiled from participants revealed that, once access was obtained, differences existed in the type of CM offered and the form of interpersonal relations developed with the FP promoters, depending on whether they were attending a regular FP or CPOE consultation.

#### “Easy” access—with normalized barriers

Although all the users analyzed in the study, by definition, had obtained some degree of access to health services (as opposed to those who did not reach the facilities), the experiences narrated in their discourses revealed the presence of barriers. The discourses of the interviewees generally classified FP access as “easy;” however, it was clear from their accounts that bureaucratic barriers had been incorporated and normalized by them as part of the critical path to health care. To mention some of the major barriers observed, those seeking FP care were required to be affiliated with the SP and show evidence of their insurance policy; otherwise, they were charged for the consultation and CM provided. They were also required to register and open a medical file, show their Health Card, complete a form, obtain an appointment through a system that required their physical presence, and endure long waiting times to obtain an admission pass and be seen by staff.

Noteworthy in the discourses of respondents were references to barriers concerning alleged health-related requirements such as bathing prior to their appointments. These access barriers were expressed by the majority of users irrespective of the purpose of their visits (e.g., general FP or CPOE) or the state where the facilities were located.

The following testimony reflects the voice of a 28-year-old IUD patient. This woman affirmed that obtaining a CM was “easy” but described a series of requirements that she was obligated to fulfill before being able to receive the device: some of these requirements were extraneous to the national health care protocols, such as being affiliated with the SP or having to bathe before consultation.

*“It’s **easy** to obtain contraceptive methods at the health center. **Depending on the contraceptive method, that’s how they ask for requirements. In the case of IUDs, they asked me for my Seguro Popular insurance policy and my Health Card. They told me to come on my second day of menstruation and to take a shower before.** To obtain it, first I go to get **a pass at 7 in the morning**, and when it’s my turn I go to gynecology and that’s where I obtain the family planning method.” (Mexico State, woman, 28 years old, married, bachelor´s degree, housewife*, *IUD, E29)*

This testimony reveals that bureaucratic barriers violating the right to access service had been normalized by the user as part of the normal health care process.

The second testimony describes the experience of a CPOE service user who reported that she had been required to attend talks offered by the *Oportunidades* human development program, currently known as *PROSPERA*. This anti-poverty program distributes resources to the underprivileged (nearly seven million Mexican families) based on a conditional cash transfer mechanism. It was designed to enhance the capacities of the poor through improved nutrition, health and education. Notwithstanding the benefits of this initiative, attending *PROSPERA* talks is not a prerequisite for obtaining a CM.

*“It was **easier** to get the methods at the health center. Now, to get contraceptive methods **the requirement is to attend the Oportunidades (PROSPERA) talks and to present the Appointment Card or the Woman’s Health Card before getting a consultation. If you don’t have the Card they don’t give you service.**” (Nuevo Leon, woman, CPOE, 45 years old, free unión, middle school, housewife*, *BTL, E01)*

The preceding testimony clearly shows that access was refused to those who did not comply with the *PROSPERA* requirement, a program with no relationship whatsoever to FP services. Nonetheless, this user perceived that access was “easy”.

Only a few users affirmed that accessing health services was difficult, with one woman from Mexico State putting it as follows:

*“**It isn’t easy**. They ask lots of irrelevant questions, and many of them don’t want you to ask them questions and you get discouraged [from coming to health services].” (Mexico State, woman, 29 years old, married, housewife*, *BTL, E24)*

#### Differential offering of CMs to regular FP users vs. CPOE patients: Offering mediated by hierarchical mechanisms

Our second finding demonstrated that, once access to services was achieved, health providers offered different methods to women presenting at the facilities for regular FP as opposed to CPOE services. While the first group was offered a variety of CMs, the second was consistently offered only the IUD method and was not provided sufficient information. Our results also showed different patterns of treatment for the two types of users, with a top-down relationship observed particularly in the cases of those attending CPOE consultation. All this undermined the ability of women to freely choose their method of contraception.

In the following testimony, it is possible to identify the wide-ranging offering made to an 18-year-old woman during a regular FP appointment.

*“[They told me] that there were various methods for when one doesn’t want to get pregnant, and [asked] if we wanted one of the methods, so that they could insert it or give us what we were going to take: the pills, the IUD, the injection, the one every month, the one for the arm (‘the implant,’ confirmed the interviewer) or the condom. They told us that the implant wasn’t available, but the others were.” (Morelos, woman, 18 years old, married, elementary school*, *housewife, IUD, E62)*

This woman was offered a wide variety of CMs to choose from. In contrast, the following testimonies demonstrate that CPOE patients were exclusively offered the IUD method. Moreover, their offerings were made with the provider exhibiting a top-down behavior and, in one case, openly imposing this method. The first testimony below was provided by a 20-year-old woman who was offered an IUD under pressure. She was urged to sign an informed consent form during delivery and was told only after childbirth that she had consented to the insertion of an IUD. In the second case, an 18-year-old woman had an IUD placed, with the health provider alleging that it was an obligatory service and offering her the freedom to decide to have it removed at a later time if she did not want it. This testimony also reveals that the patient would have preferred an implant.

*“I felt pressured when they offered me the IUD during delivery. **“I didn’t know what was going on, and the doctor told me, ‘sign, sign,’ and I said, ‘fine, I’ll sign**.**’** The next day they explained everything they did to me, **and they told me ‘you have an IUD**,**’** and I said, ‘oh, that’s alright.’” (Mexico State, woman, 20 years old, single, high school, housewife*, *IUD, CPOE, E17)*

*“[The doctor] recommended an IUD. He told me…. ‘Well, in the General Hospital **they have to put it** [the IUD] **in, it’s obligatory,** […],’ and he said that for that reason it was better to have it inserted here, **and if I didn’t want it anymore, he could remove it and put in an implant** [her preferred method] **if I wanted**.” (Morelos, woman, 18 years old, free union, middle school*, *housewife, IUD, CPOE, E68)*

### Availability of contraceptives and free choice

In general, the accounts of the users revealed a shortage in the availability and a reduced variety of preferred methods (e.g., the implant, the patch and the injection) coupled with the availability of the methods least desired (e.g., the IUD, condoms, and daily hormones).

#### Inverse availability with limited freedom of choice depending on the type of consultation

The differentiated availability of methods where those in stock at FP facilities are not the ones women prefer can be called *inverse availability*. Unable to obtain the methods they prefer or that best respond to their needs, many women are prevented from exercising their right of free choice with respect to contraception. In the case of interviewees attending CPOE consultation, pressure to choose the IUD method was clearly reflected in their discourses. In the testimony below, an 18-year-old woman explicitly affirmed that the possibility of women choosing their preferred CMs was nonexistent because selection was based on the supply available at the FP facility, thus illustrating the concept of inverse availability.

***“They don’t let you choose the method. They only tell you what’s available at that moment, and that’s what they offer.”** (Coahuila, woman, 18 years old, married, middle school, works*, *IUD, E47)*

The following testimony, too, reveals a case of inverse availability in which the CM available at the health facility did not correspond to the user’s preference. The IUD method was once again induced as a routine practice following childbirth.

*“I had been thinking of protecting myself [against becoming pregnant] but I didn’t want that one [referring to the IUD]. **I preferred the implant, but that one wasn’t offered to me**. When she [her daughter] wasn’t born yet, they told me that they were going to put it in. **I told him [the doctor] no, another method, but, well, they put it in** [an IUD].” (Morelos, woman, 20 years old, married, middle school, hosewife*, *no CM, E66)*

Here, the condition of inverse availability generally faced by women was compounded by another frequently observed condition: a differential CM offering contingent on the type of consultation requested: FP or CPOE. Requiring the second type of FP service, the above user was strongly directed towards using the IUD method.

#### The implant: One of the most preferred and requested but least available methods

Among our sample, substantial and detailed evidence showed that the implant was one of the methods most in demand but with limited availability. Shortage of this and other unavailable methods required women to wait for long periods and curtailed their freedom of choice, as can be seen in the following comment. This 24-year-old woman unequivocally expressed her preference for an implant but was given another CM due to inverse availability:

*“Last time I came here **I had already decided on an implant so I wouldn’t have to worry for three years, but they didn’t have any. This is a failure of the system because it’s supposed to be public health,** and they’re out of everything. They apologized saying that it was the end of the year*, *but for me, that’s no excuse. **They did give me an injection, but I wanted a longer-term method.”** (Mexico State, woman, 24 years old, single, bachelor’s degree, injection, E22)*

This user explicitly stated that the inability of women to select their preferred CM constituted an error on the part of the health care system.

### Interpersonal relations

Service quality was also explored from the standpoint of interpersonal relations, an important aspect of care. Users in the study have defined adequate interpersonal relations as a warm and respectful attitude coupled with the provision of satisfactory information, for instance, about HIV/AIDS and CMs including permanent alternatives.

#### Dependency relations on the part of users

While none of the users characterized the care provided as indifferent or disrespectful, their comments indicated experiences where staff appeared to have induced their contraceptive decisions. The first type of interpersonal relation described could be characterized as one of dependency. It involved a relationship where staff offered suggestions but little information to guide the users’ decision making. Providers offered FP services with a tendency to point users towards decisions that were in their opinion correct, thus leading to user dependency. The second type of interpersonal relation involved overt coercion: the staff openly induced or forced users to adopt a specific type of contraceptive. The collected discourses achieved data saturation and density with respect to this finding.

The following testimony conveys a relationship of dependency in which the provider directed the user towards using a specific mehod, and the user responded by accepting his or her recommendations without objection.

*“I decided to have an operation so that I wouldn’t have more children since I was asked during my pregnancy control consultations how I was going to avoid getting pregnant in the future. I chose to get a BTL. **I think it’s alright that they strongly encourage us to use these methods. It helps us not to have children so often.** It’s good that they pressure us…. For example, right now, they’re sending us to have mammograms. **I say that it’s good that they pressure us and, also, it’s for our own good.”** (Nuevo Leon, woman, 45 years old, free union, middle school*, *home, BTL, CPOE, E01)*

The preceding testimony contains the discourse of a FP service user who openly accepted being pressured by health providers into performing actions they recommended.

#### Relations involving nonconsensual imposition

The second case of interpersonal relations observed involved experiences ranging from insistent suggestion to the violation of the right of women to free reproductive choice. It is striking that in the vast majority of the cases recorded, whether under pressure or overt imposition, the final objective was the use of the IUD method. One consistent finding concerned the intensity of the pressure exerted during CPOE sessions, particularly in urban clinics providing birth care on a regular basis. This behavior was noted in both primary- and seconday-care facilities.

The following discourse from a 23-year-old user attending a regular FP consultation in a primary-care facility describes the pressure this woman underwent to use an IUD:

***“When my baby was born I felt pressured by the doctor to have the device put in… I wanted to learn about other methods, but they didn’t give me any other options.”** (Mexico State, woman, 23 years old*, *free union, high school, home, IUD, E33)*

In this testimony, the user described the pressure she was subjected to as well as the lack of information and options offered during CPOE consultation. In the following cases, also concerning CPOE care, the interviewees unambigously related how an IUD was placed against their will in the hospital. In the first case, a 21-year-old woman denounced the violation of her rights: the health providers forcefully inserted an IUD threatening to perform a BTL if she resisted:

*“After giving birth, **they told me at the hospital that if I didn’t accept an IUD they were going to operate on me** [to perform a BTL]. **There, they did force me** [to get an IUD]**. Afterwards, I went to the health center in Cuentepec to have it taken out. I didn’t report the fact that I had been forced.** I felt that they hadn’t respected my rights […]. (Morelos, woman, 21 years old, free union, middle school, home, injection*, *E54)*

The second testimony reflects the voice of a 22-year-old user who had an IUD inserted despite her explicit refusal to authorize the procedure:

*“I felt pressured to use a method. During delivery they didn’t put it in and they told me that they were going to do it the next day, but **I didn’t want it anymore because it hurt and I was very swollen. I told him** [the provider] **that it was better if I came later because I had a lot of pain in my stitches. And he told me no, that they were going to insert it** [an IUD] **right away… and they put it in, they didn’t take my decision into account.”** (Campeche, woman, 22 years old, married, bachelor’s degree, merchant*, *IUD, CPOE, E76)*

In this account, the user denounced not only the violation of her rights but also the use of medical violence given the pain she was expressing. These testimonies document the different types of existing provider-patient relations, ranging from user dependency marked by poorly informed consent to overt imposition and even human rights violations on the part of health providers. Both types of relations were mediated, once more, by the type of consultation requested.

### Information provided to clients

#### Scarce and erroneous information on CMs and HIV

The following testimonies clearly reflect that the unacceptable knowledge of many women concerning CMs and HIV prevention is based on information provided by FP promotors. In the first case, the user related erroneous information on the adverse health effects of several CMs and declared that she had obtained it from the health promotor at the clinic.

*“Not to use **contraceptive pills** for a long time because **they poison the body,** not to use them for more than three years. **The IUD, you have to check it every month. The implant tends to make you gain weight. You have to be thin to use it.** They gave me this information in a talk*, *it was a health promotor.” (Nuevo Leon, woman, 42 years old, married, middle school, housewife, condom, E14)*

Myths such as those replicated in the previous testimony can be reinforced by erroneous information from FP promotors. The following account of a 28-year-old woman regarding the prevention of HIV and other sexually transmitted infections (STIs) provides another example of inadequate FP information offered to users. This woman stated that monogamy constituted a method for HIV prevention:

*“**If you have relations with other people** [apart from your husband] **you can be infected** [with HIV]. This was [the information] given at the health center about seven months ago.” (Nuevo Leon, woman, 28 years old, free union, first year of middle school, home, BTL, CPOE*, *E12)*

Both of the above cases indicate that, although the providers touched on important FP issues, the information offered was inaccurate, perpetuating myths about HIV prevention and the possible side effects of CMs; the information regarding HIV prevention was alarming. Only two of the interviewees in our sample mentioned the condom as a form of prevention.

#### The case of BTL: Reports ranging from lack of information to the violation of human rights

Finally, we explored the information provided to users in relation to BTL. It should be noted that the majority of interviewees (N = 66) were under 30 years of age, which may have contributed to the failure of providers to offer information concerning permanent methods. However, those over 30 who had at least one child (N = 20) also reported not having received information on definitive methods. This is significant, as the likelihood of reported information on these methods would be expected to be highest among this group of women.

With regard to the BTL method, our findings revealed a lack of information compounded, in some exceptional cases, with interpersonal relations denoting outright user dependency towards staff. Some women who had undergone BTL reported that they had been informed only about the permanence of the method. In one case—coincidentally a woman attending puerperium consultation—BTL was performed against her will and without her being offered any information about the method. This case demonstrated unacceptable treatment of users and their families by the health care system.

The following testimonies bring to light the lack of information available to users prior to undergoing BTL:

This case features a 45-year-old woman. She had undergone BTL, with the medical staff warning her only about the possibility of experiencing acute anxiety and “nervousness” following the operation:

*“When I told him [the health professional] to go ahead and operate, **he told me not to get scared, because sometimes one could feel like desperate and get really nervous**. And I told him I didn’t think that would happen because I did want to have the operation. That was all [the information he gave me].” (Nuevo León, woman*, *45 years old, free union, middle school, housewife, BTL, CPOE, E01)*

The case of insufficient information described above pales in comparison to the event experienced by the following user. In this exceptional case, the medical staff decided to operate on a woman in puerperium despite her expressed wish not to have the operation, this on the grounds that she had already signed a consent form:

*“When they operated [BTL] on me they asked me to sign the consent form. **I regretted it afterwards, but since I had already signed there was nothing they could do** […] **I hadn’t even had the operation when I started to regret it** […] **but I had already signed the form and my husband had given his authorization, so there was nothing I could do.”** (Morelos, woman, 31 years old, married, fourth grade of elementary school, employed, BTL, CPOE*, *E55)*

This testimony depicts an unacceptable combination of events: a lack of information on the scope of informed consent, a violation of human rights and a breach of the freedom of reproductive choice within the framework of FP service delivery.

Table 2 summarizes the findings of our study on user perceptions regarding the quality of FP services in Mexico. It was elaborated according to the types of users and quality components analyzed.

**Table 2:** FP quality components. Summary of user perceptions by type of user.

FP quality components							
Type of user	Access		CM availability		Interpersonal relations	Information provided to users	
	To services	To CMs	Availability	Free choice	Respectful attitude	On CMs, HIV and STIs	On BTL
**Regular FP consultation**	“Easy” access—with normalized barriers	Offering mediated by hierarchical mechanisms; differential offering of CMs to regular FP users vs. CPOE patients	The implant, one of the most preferred and requested but least available methods	Limited freedom of choice under conditions of inverse availability	Dependency on the part of users	Scarce and erroneous (on CMs and HIV)	Scarce and erroneous
**CPOE consultation**		Offering mediated by hierarchical mechanisms; offering steered towards using the IDU IUD		Without free choice–offering of IUD only	Nonconsensual imposition; human rights violations		Reports ranging from lack of information to the violation of human rights

Source: Prepared by the author based on information gathered during interviews

## Discussion

The results of this study highlight the persistence of problems regarding access, availability, interpersonal relations and the information provided to clients as quality components of family planning (FP) services in Mexico. Despite the efforts of public health policy to strengthen FP interventions—including a large sustained increase in funding for these services—the discourses of service users clearly point to areas of opportunity for improvement.

While no significant differences were observed by geographical region or type of location (rural/urban), sharp differences emerged among types of service users, particularly between those attending regular FP appointments and those receiving consultations for purposes of contraception post obstetric event (CPOE).

### Internalized access barriers to family planning services

In general, service users felt that access was “easy” but rarely considered the levels of access that would be required to ensure an effective provision of services. They were satisfied with simply being able to utilize the services, notwithstanding the bureaucratic, organizational, economic and interpersonal hurdles experienced. Normalization of these barriers probably accounts for the high levels of satisfaction usually reported in quantitative analyses [6] despite the access barriers revealed in user testimonies. This is consistent with findings from a study in Peru by Vicuna in 2002 [49].

With respect to internalized barriers, it is important to mention the socio-demographic profile of FP users in public health facilities. They are normally women exhibiting high structural and social vulnerability–low educational and socioeconomic levels, as well as a lack of formal employment and Health Care protection–which may limit their ability to demand respect for their rights and undermine their empowerment when seeking and receiving health services. This is in line with the findings of previous studies that have indicated a lack of user objectivity in perceiving concrete service conditions within contexts exposed to amalgamated cultural and social influences [50,51].

Other barriers to access identified by our research concern the fact that those in need of FP services must pay fees unless they are affiliated with the Seguro Popular or *PROSPERA*. Our findings match those of a study conducted in Canada in 2015, where it was observed that the need to pay fees was one of the principal barriers to FP care. People lacking the capacity to pay were unable to undergo FP processes, and thus faced a greater risk of unwanted pregnancies. This was especially the case among adolescents, young adults no longer eligible to receive services at youth clinics, immigrants, and the working poor [52]. Economic barriers to FP access represent a limitation in receiving quality care.

Organizational and managerial limitations identified in the participating health facilities also require immediate attention. Institutional and administrative barriers resulting from limited service hours, excessive paperwork and long waiting time for obtaining services were evident from the testimonies presented and must be considered a major issue in the provision of FP services [53–55]. Our results are consistent with the international literature in demonstrating that bureaucratic barriers are crucial in determining whether or not users are able to benefit from health services [53–55].

### Inverse availability of contraceptive methods at family planning facilities

The discourses of the interviewees reveal that users must confront the scarcity of preferred birth control methods such as the implant, the patch and injections, and make decisions under conditions of inverse availability, that is, being forced to choose what is available rather than what they prefer. While the patch and the implant are the methods most preferred and requested by users, they are the ones least available in FP facilities. The inverse relationship between preferences and availability identified in our study undermines freedom of choice. A study by Hutchinson et al. in 2011 supports our findings that the frequent scarcity of supply and inadequacy of information on different methods can inhibit their continued use over the long term [56]. Misalignment of the contraceptive methods (CMs) available at FP facilities with those preferred by users has been previously identified in the literature [57]. In a study of Burundi, Ndayizigiye et al. (2017) reported that women preferred long-term CMs which were, however, the least available at health centers. Comparable to our results in Mexico, the implant, the method most preferred by women in Burundi, was available for only 40% of the study sample. Referring to the long-term effect of this method, the arguments of users highlighted the covenience of utilizing a contraceptive that did not require them to return to the provider for three years [57]. The Burundi study also identified what the authors designated as a “climate of fear” among users, who were afraid of being seen by others while seeking FP services. This emerged as one of the factors behind the limited use and coverage of FP services.

The issue of CM availability at FP facilities also calls for a discussion regarding the purchase and distribution mechanisms for the supply of CMs in the Mexican public health sector. Further analysis of the determinants of supply deficiencies is warranted, particularly in view of the sustained increase in public investment that maternal health and FP have enjoyed in recent years [20]. Unwanted effects ensuing from a lack of strategic planning in the acquisition of CMs (often guided by previous budgets and patterns of consumption), irregularities in the supply chain and an inefficient use of resources could be causing a problem as visible and tangible as the lack of supply in the facilities [58–60]. A reliable and responsive supply chain, providing an adequate inventory of the right contraceptives, under appropriate conditions, when and where they are needed, is key to overcoming these challenges [58–60]. Other studies have documented the importance of streamlining and standardizing the supply process as a *sine qua non* for achieving FP goals. Our results highlight deficiencies in the supply chain at the point of delivery and, consequently, in the provision of FP services to users.

### Examples of abuse and disrespect in the interpersonal relations at FP facilities, representing human, sexual and reproductive rights violations

The top-down, paternalistic and intrusive relationship between users and providers is at times so extreme in the provision of CMs that it represents a violation of the human rights of users. This is especially true in the case of women being treated for CPOE and IUD placement, constituting an important barrier to receiving quality care. According to data reported by Hulme et al. in 2015 [52], the beliefs, prejudices and preferences of providers towards certain CMs can influence their prescription. For instance, some prescribe oral CMs indiscriminately, even for women who have difficulty in taking daily pills, while others refrain from prescribing certain methods such as the IUD based on the mistaken belief that they should not be used by nulliparous women. The information analyzed in our study does not allow for proving that induced demand reflects the beliefs of providers; it does however demonstrate that it is linked to the unavailability of certain methods at the facilities. Our findings are also in agreement with those of other studies in highlighting the central role of interpersonal relations in either facilitating or deterring access to health care. Relations that do not convey a friendly attitude and quality care have been identified as a barrier for those who seek FP services [27]. The discourses of our respondents reveal unfortunate–if rare–cases of violation of human rights, preventing women from selecting their own FP methods.

Even though CPOE represents one of the most cost-effective and useful maternal health interventions, the perceptions of users in our study document the frequent lack of dignified and respectful treatment at health facilities during the procedure. Treatment is often violent: in some cases users are told that certain methods are mandatory, that they are required to return to the clinic at a later date if they wish to remove their IUD, and that they must use an IUD against their will despite their physical discomfort following childbirth.These findings suggest that training and sensitivity interventions for maternal health care providers are necessary to ensure that normative and regulated procedures are carried out with utmost respect and under the proper informed consent of users. Disrespectful treatment–at times even violent and in violation of their human rights, for instance, in those cases where women are forced to undergo a BTL procedure against their expressed will–indicates the absence of quality interpersonal care in explicit violation of the sexual and reproductive rights of users.

### Provision of inadequate or erroneous information

The function of offering information to users is an opportunity to provide them with FP counseling and accurate, evidence-based facts. This function is intended to open the possibility of dispelling myths and ensuring that users understand the information provided regarding treatment, continuity in care and the onset of side effects. It also allows FP staff to build a relationship with users conducive to sustained interaction [34]. Nonetheless, our findings indicate that providers repeatedly miss this opportnunity. This is evidenced in the accounts of our respondents regarding inaccurate and often erroneous information provided. Studies conducted in other contexts refer to the fact that that providers, acting as guardians of information, frequently prioritize what they communicate to their clients [34] and can selectively avoid subjects that they consider to be potentially embarrassing or to cause uneasiness for either themselves or their clients. This is particularly evident during the provision of sexual and reproductive health services given that sexual problems often prove difficult to broach, especially when they involve behavior regarded as socially inappropriate or taboo [34].

### Limitations

Our findings apply only to users of services provided by the Ministry of Health, and thus cannot be generalized to women using Health Care protection services. Since interviews were conducted solely with users encountered in public health facilities, the present study cannot address barriers to access for users of other services. While qualitative findings can be transferable to populations and circumstances similar to those analyzed, they do not have the potential to be generalized.

In spite of our efforts to protect user confidentiality, the fact that interviews took place in health facilities may have contributed to the underreporting of testimony critical of the poor quality of services.

Although the study included rural areas, our field staff spoke no indigenous languages and therefore concentrated on mestizo women. We do not know what additional barriers are confronted by indigenous women in accessing quality services.

### Strengths

We employed various mechanisms to ensure the rigor and validity of our qualitative analysis, and hence, the robustness of our findings.

While we sought to obtain a broad panorama by sampling six culturally diverse states including urban and rural areas with different levels of marginalization, we cannot guarantee that all our results are applicable nationally. Nevertheless, specifically with regard to CPOE, given the saturation and density of our data and the fact that the reported conditions were encountered in all of the facilities analyzed, it can be concluded that our findings are transferable [61] to similar situations throughout Mexico.

## Conclusions

After six decades of FP service supply, our results suggest a series of quality issues that may lie at the heart of the unmet demand reported in the literature. Based on a comprehensive conceptual scheme, the present study analyzes the quality of FP services, highlighting areas for improvement that should be considered by the MoH on Mexico in future efforts. In sum, our study reveals sizable and unacceptable barriers to obtaining quality FP services in a country whose efforts to improve both coverage and quality of such services have been constant and deliberate [62]. Quality maternal and infant health care is without a doubt a pillar of national development and is of paramount importance for health care systems.

## Supporting information

S1 Fig Dimensions for evaluating the quality of family planning services. Source: Elaborated by the authors based on the conceptual frameworks of Bruce-Jain [29–31], Creel [26] and Donabedian [33].
(TIF)

S1 File Guide for interviews in Spanish. (PDF)

S2 File Guide for interviews in English. (PDF)
