# Supplementary figures and images for: Quality of family planning services in Mexico: The perspective of demand
Source: PLoS One. 2019 Jan 30;14(1):e0210319. doi: 10.1371/journal.pone.0210319 (PMC6353096; doi:10.1371/journal.pone.0210319)

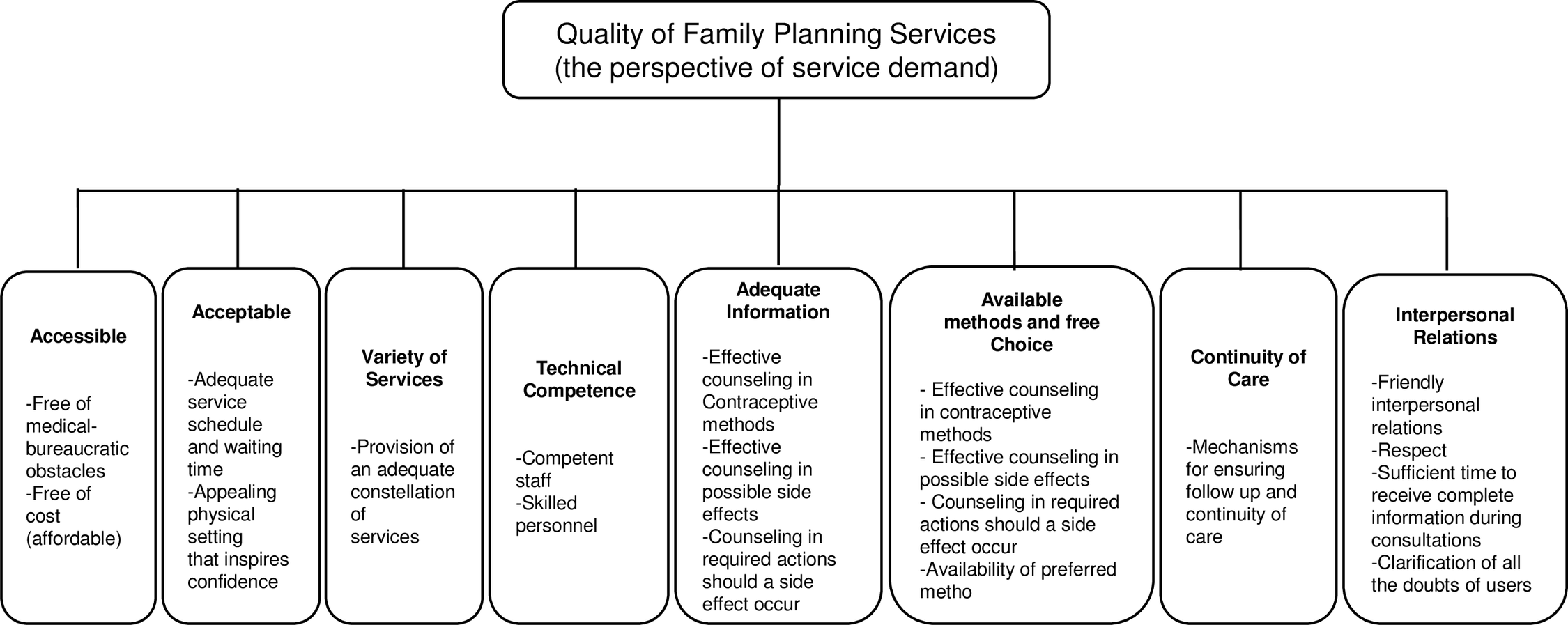

Supplement: S1 Fig — Source: Elaborated by the authors based on the conceptual frameworks of Bruce-Jain [29–31], Creel [26] and Donabedian [33]. (TIF) [file pone.0210319.s001.tif]
