# Supplementary material for: Quality of family planning services in Mexico: The perspective of demand
Source: PLoS One. 2019 Jan 30;14(1):e0210319. doi: 10.1371/journal.pone.0210319 (PMC6353096; doi:10.1371/journal.pone.0210319)
Supplement: S1 File — (PDF) [file pone.0210319.s002.pdf]

Instituto Nacional de Salud Pública

Centro Nacional de Equidad de Género y Salud Reproductiva

Organización Panamericana de la Salud
Oficina Regional de la Organización Mundial de la Salud

“Estudio para la determinación de la situación actual del Programa de Planificación Familiar y Anticoncepción como línea base para su comparación con evaluaciones del desempeño”

Guía de entrevista para usuarias(os)

IDENTIFICACIÓN DE LA ENTREVISTA
Lugar de la entrevista: ____ Fecha: ____________
Hora: ________________ Sexo: (F) (M) Iniciales del entrevistado: ____________________
Tiempo aproximado de duración de la entrevista: _____________________________
Nombre de la institución: ___________________________________

Objetivo de la entrevista

Indagar sobre la disponibilidad y uso de los métodos anticonceptivos, así como la orientación, consejería y difusión de los diferentes métodos anticonceptivos recibidos en las unidades de salud.

Elementos a explorar

A. Datos generales, edad, sexo, estado civil, nivel de estudios, etc.
B. Acceso a servicios de Planificación Familiar
C. Conocimientos, uso, acceso e información de métodos anticonceptivos y servicios de PF

Introducción a la entrevista (posterior a la lectura del consentimiento oral)

Buenos días, mi nombre es (nombre de la entrevistadora) y trabajo en el Instituto Nacional de Salud Pública. Como le comenté mis compañeras y yo estamos realizando algunas entrevistas a quienes como usted, son actores clave en la implementación del programa de Planificación Familiar, constituyendo un elemento clave de la demanda de métodos anticonceptivos.

La finalidad de esta entrevista es que me cuente cuál es su experiencia con el programa de Planificación Familiar, el tipo de servicios que ha utilizado, así como escuchar sus opiniones y sugerencias sobre el funcionamiento del programa.

GUÍA DE PREGUNTAS

A. Datos generales, edad, estado civil, nivel de estudios, etc.

1.- Por favor, ¿me podría decir qué edad tiene?

2.- ¿Cuál es su estado civil?

3.- ¿Cuál es su nivel máximo de estudios?

4.- ¿Actualmente trabaja? ¿Cuál es su actividad principal?

B.- Acceso a servicios de Planificación Familiar

1.- ¿Por qué razón decidió acudir a los servicios de planificación familiar?

2.- ¿Fue fácil acceder a los servicios de planificación familiar?

3.- ¿Cómo obtuvo su cita? ¿Cuál es el mecanismo para obtener una cita en los servicios de PF en esta unidad?

C.- Conocimientos, uso, acceso e información de métodos anticonceptivos

Conocimientos sobre métodos anticonceptivos

1.- ¿Cuáles son los métodos anticonceptivos que usted conoce?

2.- ¿Conoce usted algún problema para usar métodos anticonceptivos? ¿Cuáles son? ¿Qué métodos dan esos problemas?

3.- ¿Conoce o ha escuchado de la pastilla anticonceptiva del día siguiente? ¿Qué es lo que ha escuchado? ¿Conoce en qué situaciones debe utilizarse?

Uso previo de métodos anticonceptivos

4.- ¿Ha utilizado alguna vez métodos anticonceptivos? No ¿Por qué? → En caso de una respuesta negativa pase a la pregunta 23

5.- ¿Qué métodos anticonceptivos ha utilizado? ¿Desde cuándo?

6.- ¿Quién le sugirió que utilizara estos métodos anticonceptivos? ¿Cómo los obtenía?

7.- ¿Por qué razón o con qué finalidad decidió que utilizaría métodos anticonceptivos?

8.- ¿Le resultó difícil obtenerlos? ¿Estuvieron siempre disponibles? En caso de no estar disponibles en su centro de salud, ¿cómo los obtenía?

Uso actual de métodos anticonceptivos

9.- ¿Actualmente utiliza algún método anticonceptivo? ¿Cuál? ¿Desde cuándo?

10.- ¿Cómo obtiene el método anticonceptivo que actualmente utiliza?

11.- ¿Siempre está disponible el método que usted utiliza?

12.- ¿En caso de no estar disponible, qué le recomiendan hacer?

13.- ¿Alguna vez ha necesitado la pastilla anticonceptiva de emergencia?

14.- ¿Ha utilizado la pastilla anticonceptiva de emergencia?

Acceso a los métodos anticonceptivos

15.- ¿Considera que es fácil acceder a los métodos anticonceptivos en el centro de salud? Explique

16.- ¿Qué requisitos le pidieron la primera vez que solicitó algún método anticonceptivo?

17.- ¿Usted sabe si las personas pueden acudir libremente a los servicios de PF cuando sientan que tienen problemas con los métodos anticonceptivos? Explique

18.- ¿Ha solicitado la pastilla anticonceptiva de emergencia en su centro de salud? ¿Fue fácil obtenerla?

19.- ¿Se ha sentido presionada(o) por el personal de salud para utilizar algún tipo de método anticonceptivo?

20.- Cuando ha solicitado algún método anticonceptivo, ¿han tomado en cuenta su preferencia por algún método en particular? ¿Le han dado la oportunidad de elegir?

21.- ¿El lugar donde se le otorgan los métodos anticonceptivos es cómodo y cuenta con la suficiente privacidad para que usted este cómoda (o)? Explique

Información sobre los diferentes métodos anticonceptivos

22.- ¿Los servicios de planificación familiar le ofrecieron información sobre los diferentes métodos anticonceptivos que existen? ¿Qué información le dieron?

23.- ¿Qué personal del centro de salud fue el que le dio la información sobre métodos anticonceptivos?

24.- ¿En la información que recibió le hablaron sobre las ventajas, desventajas, contraindicaciones y posibles daños a la salud por el uso de los diferentes métodos anticonceptivos?

25.- ¿El personal que le dio la información se aseguró que a usted le quedara muy clara toda la información sobre las ventajas, desventajas, contraindicaciones y posibles daños a la salud por el uso de los diferentes métodos anticonceptivos?

26.- ¿En caso de que se haya decidido por un método anticonceptivo permanente (como OTB o vasectomía)? ¿Qué orientación se le dio y cuántas veces? ¿En qué unidad u hospital le ofrecieron el servicio? ¿Firmo usted un consentimiento sobre el procedimiento a realizar?

27.- ¿La información, que ha recibido, en la consulta de planificación familiar incluyó temas sobre infecciones de transmisión sexual y VIH/SIDA?

28.- ¿En el centro de salud existe información y/o promoción de los servicios que brinda el programa de planificación familiar?

29.- ¿La información sobre los tipos y uso de métodos anticonceptivos se imparten de forma regular tanto a hombres como a mujeres de cualquier?

30.- ¿Se respetaba la decisión de los usuarios de aceptar o no un método anticonceptivo?

31.- ¿Les permiten elegir libremente sobre el número y espaciamiento de sus hijos y del método anticonceptivo que mejor cumpla con sus necesidades?

D.- ¿Le gustaría comentar algo más?

¡Muchas gracias por su participación!
