# Supplementary material for: Quality of family planning services in Mexico: The perspective of demand
Source: PLoS One. 2019 Jan 30;14(1):e0210319. doi: 10.1371/journal.pone.0210319 (PMC6353096; doi:10.1371/journal.pone.0210319)
Supplement: S2 File — (PDF) [file pone.0210319.s003.pdf]

Instituto Nacional de Salud Pública

Centro Nacional de Equidad de Género y Salud Reproductiva

Organización Panamericana de la Salud
Oficina Regional de la Organización Mundial de la Salud

# “Study aimed at determining the status of the Family Planning and Contraception Program as a baseline for comparison with performance evaluations”

## Guide for interviews with users:

INTERVIEW IDENTIFICATION

Place of the interview: ________________________________________ Date: ____________

Time: ________________ Sex: (F) (M) Initials of the interviewee: ____________________

Approximate time of the interview: _____________________________

Name of the institution: ___________________________________

### ***Objective of the interview***

To inquire about the availability and usage of contraceptive methods, the orientation and counseling offered to users and the dissemination of the different contraceptive methods provided at family planning facilities.

### ***Elements to be explored***

A. General information, age, sex, marital status, educational level, etc.
B. Access to Family Planning Services
C. Knowledge, use, access and information on contraceptive methods and family planning services

***Introduction to the interview*** (after reading the oral consent to the user)

Good morning. My name is (name of the interviewer), and I work at the National Institute of Public Health. As I said before, my colleagues and I are conducting interviews with people like you,

who are key actors in the implementation of the Family Planning Program and therefore crucial players in the demand for contraceptive methods.

The purpose of this interview is for you to tell me about your experience with the Family Planning Program and the type of services you have used. I would also like to hear your opinions and suggestions regarding the way the Program works.

*QUESTION GUIDE*

## A. General information, age, marital status, educational level, etc.

1. - Could you please tell me your age?

2. - What is your marital status?

3. - What is your maximum level of studies?

4. – Are you currently working? What is your main activity?

## B.- Access to Family Planning Services

1. - What made you decide to seek family planning services?

2. - Was it easy to access Family Planning Services?

3. - How did you get an appointment? What is the mechanism for obtaining an appointment for Family Planning Services in this health care facility?

## C.- Knowledge, use, access and information about contraceptive methods

***Knowledge about contraceptive methods***

1.- What contraceptive methods do you know?

2.- Do you know of any problems related to the use of contraceptive methods? What are they? What are the methods that cause them?

3.- Do you know or have you ever heard about the morning-after pill? What have you heard about it? Do you know what situations it is used for?

***Previous use of contraceptive methods***

4.- Have you ever used a contraception method? No. Why? → If the answer is negative, skip to Question 23.

5.- What contraceptive methods have you used? Since when?

6.- Who suggested that you use those contraceptive methods? How did you obtain them?

7.- Why or for what purpose did you decide to use contraceptive methods?

8.- Was it difficult to obtain them? Were they always available? In those cases where they were not available at your health care facility, how did you get them?

***Current use of contraceptive methods***

9.- Are you currently using a contraceptive method? Which one? Since when?

10.- How do you obtain the contraceptive method you are currently using?

11.- Is the method you are using always available?

12.- In those cases where it is not available, what do the family planning staff recommend you do?

13.- Have you ever needed the emergency contraceptive pill?

14.- Have you used the emergency contraceptive pill?

***Access to contraceptive methods***

15.- Do you find it is easy to access contraceptive methods in your health care facility? Please explain.

16.- What requirements did the health care facility ask for the first time you requested a contraceptive method?

17.- Do you know whether people can visit the health care facility freely for family planning services if they feel they are having problems with their contraceptive methods? Explain.

18.- Have you ever requested the emergency contraceptive pill in your health care facility? Was it easy to obtain?

19.- Have you ever felt pressured by the health personnel to use a specific contraceptive method?

20.- The times that you have requested a contraceptive method, has the health personnel taken into account your preference for a particular method? Have they given you the opportunity to choose?

21.- Is the place where you are provided contraceptive methods comfortable and sufficiently private for you to feel at ease? Explain.

***Information about different contraceptive methods***

22.- Did the Family Planning Services offer you information on the different existing contraceptive methods? What information did they give you?

23.- Which staff members gave you the information on contraceptive methods?

24.- Among the information received, were you told about the advantages, disadvantages, counter-indications and possible side-effects to your health related to the use of the different contraceptive methods?

25.- Did the personnel who gave you the information make sure that you understood very clearly all the information on the advantages, disadvantages, counter-indications and possible side-effects to your health related to the use of the different contraceptive methods?

26.- In case of having decided to use a permanent contraceptive method (such as BTL or a vasectomy), what orientation were you given and how many times? In which health care facility or hospital did you receive the service? Did you sign a consent form for the procedure that was going to be performed?

27.- Has the information you have received during family planning consultations included themes related to sexually transmitted infections and HIV/AIDS?

28.- Is there any information or promotion in the health care facility for the services provided under the Family Planning Program?

29.- Is information on the types and uses of contraceptive methods offered regularly to both men and women?

30.- Was your decision to accept or not accept a contraceptive method respected?

31.- Are they allowed to freely choose the number and spacing of their children and the contraceptive method that best fits their needs?

31.- Does the health personnel allow you to freely choose the number and spacing of your children as well as the contraceptive method that best suits your needs?

## D.- Would you like to comment on anything else?

**Thank you very much for your participation**!
